# Circulating Tumor DNA Testing for Minimal Residual Disease and Its Application in Colorectal Cancer

**DOI:** 10.3390/cells14030161

**Published:** 2025-01-22

**Authors:** Oluseyi Abidoye, Daniel H. Ahn, Mitesh J. Borad, Christina Wu, Tanios Bekaii-Saab, Sakti Chakrabarti, Mohamad Bassam Sonbol

**Affiliations:** 1 Mayo Clinic Cancer Center, Phoenix, AZ 85054, USA; ahn.daniel@mayo.edu (D.H.A.); borad.mitesh@mayo.edu (M.J.B.); wu.christina@mayo.edu (C.W.); bekaii-saab.tanios@mayo.edu (T.B.-S.); 2 University Hospital Seidman Cancer Center, Cleveland, OH 44106, USA; sakti.chakrabarti@uhhospitals.org

**Keywords:** circulating tumor DNA, colon cancer, colorectal cancer, minimal residual disease, adjuvant chemotherapy

## Abstract

Colorectal cancer (CRC) represents a heterogeneous group of diseases that imposes a considerable global and national health burden. Although most CRC patients are diagnosed at an early stage and undergo potentially curative treatment, a significant proportion experience recurrence. Currently, adjuvant chemotherapy decisions are primarily based on clinicopathological characteristics, which have well-recognized limitations in accurately identifying patients harboring minimal residual disease (MRD), often resulting in unnecessary chemotherapy exposure. Circulating tumor DNA (ctDNA) has emerged as a promising surrogate marker for MRD, offering a more precise approach to identifying patients at risk of recurrence after curative-intent surgery and refining adjuvant chemotherapy decisions. Growing evidence from multiple studies has demonstrated that ctDNA outperforms traditional clinicopathological factors as a marker for MRD. This review synthesizes key studies supporting the role of ctDNA in MRD detection for CRC patients and evaluates clinical trials investigating the application of ctDNA in guiding adjuvant therapy decisions. This emerging strategy holds the potential to transform the adjuvant treatment paradigm in colorectal cancer by optimizing therapeutic precision and minimizing unnecessary treatment.

## 1. Introduction

Colorectal cancer (CRC) ranks as the third most common cancer and a leading cause of cancer-related deaths worldwide [1]. In addition, it plays a significant role in the global healthcare burden, with CRC accounting for 10% of global cancer incidence [2]. By 2040, the global number of new colorectal cancer (CRC) cases is expected to reach 3.2 million. In the United States alone, an estimated 152,810 new cases of CRC will be diagnosed annually, with approximately 53,010 deaths resulting from the disease each year [1]. Interestingly, the increase in CRC incidence is seen in the younger populations, with patients presenting with a mostly early-stage nonmetastatic disease that is amenable to curative intent [1].

The standard of care for patients with stage III or high-risk stage II colon cancer is surgical resection followed by adjuvant chemotherapy (ACT) based on clinical and pathological risk factors [3,4]. Despite these treatment options, 30–50% of patients develop disease recurrence [5]. In addition, another challenge is the heterogeneity with current TNM staging, leading to limitations in deciding patients who would benefit from chemotherapy, which potentially can lead to overtreatment or undertreatment in a considerable number of patients. Currently, NCCN guidelines recommend ACT for patients with resected stage III colon adenocarcinoma who are appropriate candidates for chemotherapy [4]. However, the 5-year disease-free survival (DFS) rates can differ greatly between subgroups, ranging from 89% in the lowest-risk stage III group (T1N1a) treated with surgery alone, to 31% in the highest-risk group (T4N2b) who undergo both surgery and chemotherapy [6,7]. These results imply that a considerable number of patients with low-risk stage cancer could potentially defer ACT or be eligible for a less intensive treatment approach as their outcomes are already favorable with surgical resection only. Based on this, there is a need for accurate tools beyond current clinicopathological features to identify the minimal residual disease (MRD), revise treatment selection strategy for ACT uses, and influence treatment de-escalation efforts.

**Figure 1:**
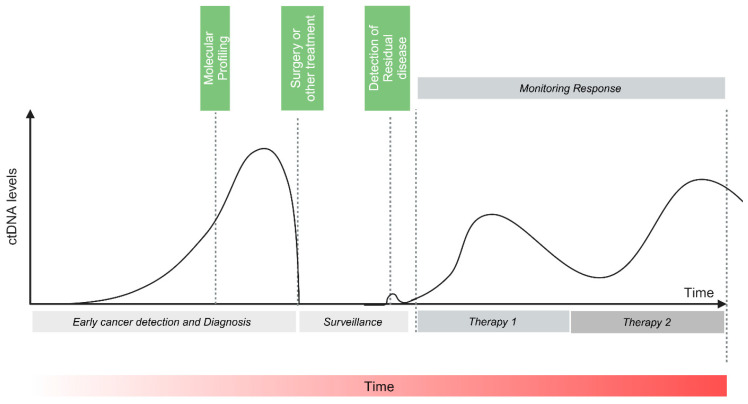
ctDNA levels in early and late stages of solid tumors. Adapted from Wan et al. [22]

Circulating tumor DNA (ctDNA) is a promising tool that could address the unmet need for a biomarker-driven diagnostic test capable of identifying MRD and revising treatment strategies for patients with CRC. ctDNA serves as a minimally invasive biomarker, facilitating the assessment of disease status across diverse cancer types [8,9,10,11]. It has many potential clinical applications at each cancer stage management. In early-stage setting, it is a valuable tool in tumor molecular profiling [8,11,12,13] and in the detection of MRD [14,15,16]. In the advanced setting, it is a valuable tool for identifying actionable genomic alterations, assessing response to treatment and identifying resistance mutations along with tumor molecular profiling [17,18,19,20,21] (Figure 1). ctDNA can potentially be a valuable tool in guiding treatment decisions for patients who have received curative-intent surgery or completed adjuvant therapy.

Numerous studies have evaluated the role of ctDNA analysis for patients with early-stage CRC and reported that ctDNA positivity following curative-intent surgery is linked to an increased risk of cancer recurrence. This review article highlights the advancements and studies evaluating ctDNA-based MRD assessment in colon cancer.

## 2. Circulating DNA

**Figure 2:**
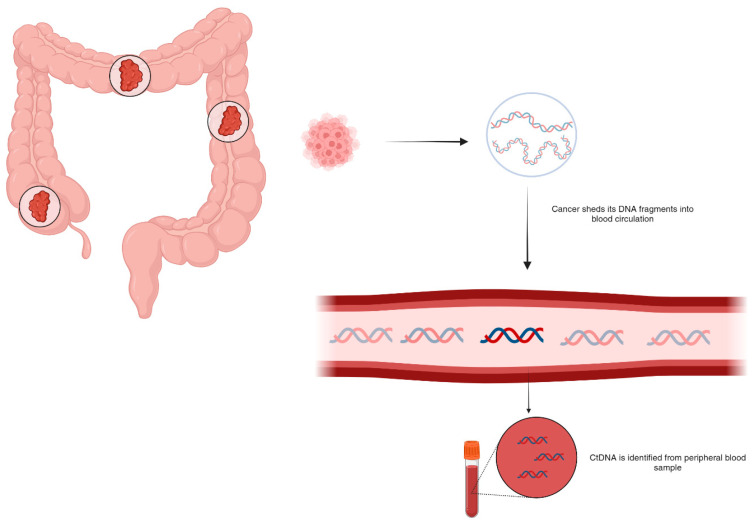
Detection of ctDNA shed in bloodstream during cancer diagnosis.

Circulating cell-free DNA (cfDNA) refers to DNA fragments that freely circulate in blood plasma, outside of cells. cfDNA is derived from normal and tumor cells undergoing apoptosis or necrosis [23]. Healthy individuals have low plasma cfDNA levels derived from hematopoietic cell turnover [24]. On the other hand, ctDNA refers to cell-free DNA fragments shed into the bloodstream by tumor cells (Figure 2). They comprise DNA fragments typically ranging from about 130–150 base pairs (bp) produced from tumor cells through apoptosis, active secretion, or tumor necrosis [22,25]. They carry the same somatic genomic alterations, mutations, and epigenetic profiles as the tumor [26]. The levels of ctDNA exhibit significant variability, with a median ctDNA fraction of 10% in metastatic cancers and less than 0.1% in early-stage cancers [22]. Elevated ctDNA levels have been associated with advanced tumor stage, increased disease burden, and enhanced tumor shedding, reflecting underlying heterogeneity [27,28].

Due to chromosomal shattering in cancer, ctDNA tends to have shorter DNA fragments, as low as 10 to 150 (bp), compared to normal cfDNA fragments, which typically measure around 160–170 (bp), the difference in fragment size allows for improved detection and analysis [29,30]. ctDNA has a reported half-life of 16 min to 2.5 h [27,31,32] and is mainly cleared by the liver with hepatocytes via phagocytosis and, to a lesser extent, through renal excretion [33]. The short biological half-life of ctDNA allows for real-time monitoring of tumor dynamics, as it rapidly reflects changes in tumor status. Additionally, the brief half-life necessitates frequent serial sampling to track ctDNA fluctuations accurately.

Analysis of ctDNA at a single time point can aid in selecting targeted therapies by providing genetic information from both primary and metastatic lesions, thereby offering insights into clonal heterogeneity and tumor evolution [10,34]. Unlike tumor biopsies, which are invasive and may not fully capture tumor heterogeneity and evolution, ctDNA analysis allows for a non-invasive, repeated assessment of a patient’s genomic tumor profile [35]. However, the optimal approach for ctDNA quantification has yet to be established. Most current assays rely on measuring somatic variant allele frequency (VAF) which is a mutation-dependent method that detects mutations in plasma at allele fractions below 0.1% [36,37]. Nevertheless, some patients might not exhibit detectable somatic variants in ctDNA, due to factors such as limited assay sensitivity, low tumor burden, or the absence of targetable somatic alterations [38]. To enhance ctDNA detection, current strategies focus on enhancing sequencing depth and incorporating various error-correction techniques [22].

## 3. ctDNA Assays

**Figure 3:**
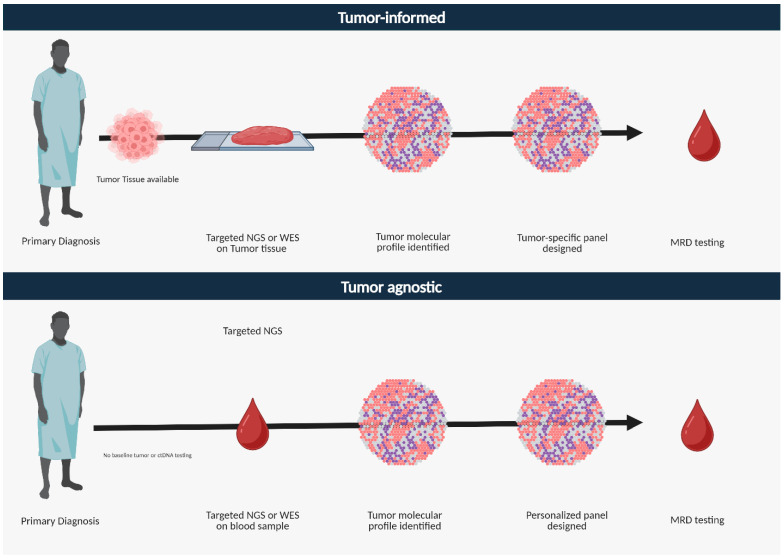
Tumor-informed and tumor-agnostic ctDNA assays for MRD testing.

Currently, two different approaches are involved in detecting ctDNA in colon cancer for MRD detection: tumor-informed and tumor-agnostic assays (Table 1) (Figure 3). The tumor-agnostic or uninformed approach involves using a fixed broad-based panel with next-generation sequencing using hotspot alterations and aberrant DNA methylation signatures known to occur in a particular tumor type while sparing the need for tumor tissue sequencing [39]. DNA methylation is one of the earliest events in CRC carcinogenesis, and incorporating methylation markers enhances ctDNA detection sensitivity [40]. This approach enables the detection of novel unexpected mutations, potentially providing the opportunity to detect MRD even after tumor clonal evolution. An added advantage to the tumor-agnostic approach is its quick turnaround time and the ability to perform the test even when tumor tissue is unavailable for testing. However, a potential drawback from tumor-agnostic approaches has been lower sensitivity and specificity for MRD detection [36,41]. A previous challenge of the tumor-agnostic approach was its vulnerability to false positives due to clonal hematopoiesis of indeterminate potential (CHIP). CHIP is a common age-related phenomenon in which hematopoietic stem cells with somatic mutations undergo abnormal clonal expansion [42,43] and has been reported to be prevalent in about 30% of cancer patients [44]. CHIP may contribute to false positives in ctDNA-based tumor genetic variant detection, particularly when non-standard variants are identified in plasma [42]. This issue is pertinent when the ctDNA mutant allele fraction is low, such as in the detection of MRD. However, advancements in tumor-agnostic assays are designed to exclude CHIP-derived mutations, thereby enhancing specificity for MRD detection [39]. In terms of application, the tumor-agnostic approach can be used in both early-stage and advanced or metastatic diseases [39].

Conversely, the tumor-informed approach leverages knowledge of tumors’ frequent hotspot mutations and variants of the index patient. This approach involves whole-exome or whole-genome sequencing or targeted sequencing of the tumor tissue [16,45]. The probes for ctDNA testing in plasma are constructed based on the patient-specific tumor genomic information. Tumor-informed assays can also be developed using a droplet digital PCR (ddPCR) platform where ddPCR partitions PCR reactions into droplets, each containing a single DNA fragment. These droplets are then analyzed using DNA PCR probes to detect target sequences. The advantages of the tumor-informed approach are its excellent sensitivity in detecting mutant alleles down to 0.1–0.01% frequency [45] in addition to its low false-positive rates [42]. The limitations of the tumor-informed approach are that it requires a longer turnaround time, higher cost implication, need for the tumor tissue, and the potential for false negatives due to tumor heterogeneity [22,28]. The tumor-informed approach is primarily used in early-stage cancers to detect MRD after curative-intent therapy (Table 1).

## 4. ctDNA-Based Detection of MRD and Prognostication in Colorectal Cancer

One of the initial studies evaluating MRD in CRC using ctDNA was conducted by Diehl et al. [27], which demonstrated that ctDNA could serve as a reliable surrogate marker for MRD in patients with resected CRC [27]. In this study, serial ctDNA assessments were performed on plasma samples from eighteen patients with metastatic CRC and oligometastatic liver disease undergoing resection, both before and after surgery. Although ctDNA levels decreased post surgery, they did not drop to undetectable levels in most cases. Postoperative plasma samples were analyzed in twenty instances, and ctDNA remained detectable in sixteen cases. Recurrence occurred in all but one of these sixteen patients. Conversely, no recurrence was observed in the four patients who did not have detectable ctDNA. This was one of the studies that established the foundation for the emerging paradigm of ctDNA-based MRD detection in CRC.

### 4.1. Studies Using Tumor-Informed Approach

Numerous subsequent studies following Diehl et al. investigated the role of ctDNA as a surrogate marker for MRD in patients with CRC (Table 2). Tie and colleagues published multiple studies that analyzed the utility of ctDNA for MRD in CRC patients. All their studies used a tumor-informed approach by performing targeted sequencing for tumor-specific genomic alterations using Safe-SeqS assay [16].

The first paper on this topic was published in 2016 [16]. This multicenter, prospective biomarker study involved 230 patients with resected stage II colon cancer, enrolled between July 2011 and September 2014, with the primary aim of investigating the potential of postoperative ctDNA analysis as an indicator of MRD. Serial plasma samples were collected for ctDNA testing 4 to 10 weeks after surgery and every three months for two years. The study detected postoperative ctDNA in 14 out of 178 patients (7.9%) who did not receive chemotherapy [16]. Patients with postoperative ctDNA-positive status exhibited significantly lower recurrence-free survival (RFS) than those with ctDNA-negative status (HR, 18; *p* < 0.001). The three-year RFS rates in the ctDNA-positive group and ctDNA-negative group were 0% and 90%, respectively, indicating patients with postoperative ctDNA-positivity had worse RFS compared to patients with ctDNA negativity. The impact of ACT on ctDNA status was assessed both during and after treatment. Among the 52 patients who received ACT, 11% (6/52) had detectable ctDNA postoperatively. Of these, 84% (5/6) had their ctDNA status changed from positive to negative during the initial phase of treatment. Notably, two of the patients became ctDNA positive following the completion of chemotherapy, both of whom later developed radiographic recurrence, while the other three remained ctDNA negative following completion of treatment. Overall, ctDNA positivity immediately after ACT completion was linked to significantly poorer RFS compared to ctDNA negativity post chemotherapy (HR 11; *p* = 0.001) [16], indicating patients who were ctDNA positive after completing ACT had worse outcomes.

When comparing ctDNA with carcinoembryonic antigen (CEA) monitoring for sensitivity in predicting subsequent radiologic recurrence, ctDNA was detected more frequently at the time of recurrence (85% vs. 41%; *p* = 0.002), indicating ctDNA was more sensitive than CEA for predicting radiologic recurrence. In addition, the prognostic significance of postoperative ctDNA status was consistent in both patients with pathologically low- (HR, 28; 95% CI, 8.1 to 93) and high-risk features (HR, 7.5; 95% CI, 2.6 to 22), indicating that ctDNA detection outperformed traditional clinicopathological risk factors in identifying patients at high risk of recurrence.

This prognostic property of tissue-informed ctDNA analysis (Safe-SeqS assay) was further studied in patients with stage III colon cancer [15]. In a study of 100 patients with resected stage III colon cancer, postoperative ctDNA was detected in 20 out of 96 patients (21%) and was associated with inferior RFS compared to those with ctDNA-negative status (HR, 3.8; *p* < 0.001) [15]. Similar to what was seen with stage II patients, postsurgical ctDNA status was independently associated with a recurrence-free interval (RFI) after adjusting for known clinicopathologic risk factors (HR, 7.5; *p* < 0.001). Additionally, ctDNA was detected in 13 out of 78 patients (17%) following chemotherapy and among those who completed the full 24 weeks of chemotherapy, ctDNA was detected post therapy in 10 out of 66 patients (15%). Patients with detectable ctDNA after chemotherapy had a higher risk of recurrence compared to those with undetectable ctDNA. The estimated 3-year RFI was 30% for patients with detectable ctDNA and 77% for those without detectable ctDNA (HR 6.8; *p* < 0.001), indicating that positive ctDNA status was associated with a poorer RFI. Conversely, patients who were ctDNA negative following surgery and remained ctDNA negative after chemotherapy demonstrated a superior RFI compared to those whose ctDNA became detectable post chemotherapy (HR, 6.5; *p* < 0.001). These findings highlight ctDNA analysis post surgery as a valuable prognostic marker for stage III colon cancer and a real-time indicator of ACT efficacy. Similar findings were noted in other studies conducted by Tie et al. in patients with nonmetastatic colon cancer and locally advanced rectal cancer (LARC) who received multimodality treatment [46,47,48].

Other studies using a tumor-informed approach (Signatera) further solidified the role of ctDNA-guided MRD assessment. One such study, conducted by Reinert et al., was a prospective investigation that included 125 patients with stage I to III CRC [45]. Patients were treated with curative surgery and provided ACT according to the institutional standard; 795 plasma samples were collected longitudinally, at baseline (up to 14 days before surgery), 30 days postoperatively, and subsequently every three months until death, study withdrawal, or 3-year period. Preoperatively, ctDNA was detectable in 88.5% (108/122) of patients, with 40% sensitivity in stage I, 92% in stage II, and 90% in stage III disease, outperforming CEA, which was detected in only 43.3% (53/122) of patients. Following definitive treatment, 10 out of 94 (10%) patients had detectable ctDNA in the 30-day postoperative period, with ctDNA analysis identifying 14 out of 16 relapses (87.5%). Overall, patients who were ctDNA positive on postoperative day 30 were seven times more likely to experience relapse compared to ctDNA-negative patients (HR, 7.2; 95% CI, 2.7–19.0; *p* < 0.001). Similarly, ctDNA-positive patients had a 17.5-fold higher risk of relapse shortly after ACT (HR, 17.5; 95% CI, 5.4–56.5; *p* < 0.001) and a 43.5-fold higher risk during surveillance (HR, 43.5; 95% CI, 9.8–193.5; *p* < 0.001) compared to ctDNA-negative patients. In multivariate analyses, ctDNA status remained an independent factor associated with recurrence after accounting for known clinicopathologic risk factors. Furthermore, serial ctDNA assessments revealed disease relapse up to 16.5 months ahead of standard-of-care radiologic imaging (mean, 8.7 months; range, 0.8–16.5 months). Lastly, serial ctDNA analysis during surveillance after definitive treatment of patients with longitudinally collected plasma samples identified relapse with 88% sensitivity and 98% specificity. Similarly, the other study using the Signatera platform [49] and studies employing ddPCR-based assays reported consistent findings [50,51].

Consistent with the above-mentioned studies, the Japanese GALAXY study showed the prognostic value of tumor-informed Signatera assay after surgery in CRC [52]. This was a large prospective, observational study that involved patients with stage II-IV resectable CRC. In this study, a total of 1039 patients were included from June 2020–April 2021. Blood samples were taken prior to surgery, one month afterward, and subsequently every three months for a two-year period, with a median follow-up period of 16.7 months. Of the 1039 patients, 18% (187/1039) tested positive for ctDNA four weeks following surgery. Out of the 187 patients with ctDNA positivity four weeks after surgery, 61.4% (115/187) experienced recurrence, whereas 9.5% (81/852) of patients with ctDNA negativity four weeks after surgery experienced recurrence (HR, 10.0, 95% CI 7.7–14.0, *p* < 0.0001). The 18-month DFS was significantly lower for ctDNA-positive patients, at 38.4% (95% CI: 31.4–45.5%), compared to 90.5% (95% CI: 88.3–92.3%) for ctDNA-negative patients, with an overall sensitivity of 63.6%. This was consistent across all pathological stages. Furthermore, in multivariate analysis for DFS in patients with pathological stage II–III disease, ctDNA positivity 4 weeks after surgery was the most significant prognostic factor associated with increased risk for recurrence (HR 10.82, 95% CI 7.07–16.6, *p* < 0.001). The GALAXY study also highlighted the benefit, or lack thereof, of chemotherapy across different CRC stages based on the MRD status. Patients with high-risk stage II/III disease who were ctDNA positive four weeks after surgery showed a significant benefit from ACT with an adjusted HR of 6.59 (95% CI 3.53–12.3, *p* < 0.001). Multivariate analysis further confirmed this finding in ctDNA-positive patients with stage II-IV disease, highlighting the absence of ACT as the strongest negative prognostic factor (adjusted HR 5.03, 95% CI 3.17–8.0, *p* < 0.001). Finally, Galaxy reported the impact of ACT on ctDNA clearance and DFS. ACT was linked to a greater likelihood of ctDNA clearance, with an estimated 68.5% of patients achieving clearance by six months following surgery, according to the analysis. For MRD-positive patients treated with ACT, those who did not reach ctDNA clearance had a markedly lower DFS and were 11 times more likely to experience cancer recurrence compared to those who achieved ctDNA clearance (adjusted HR 11, 95% CI 5.2–23.0, *p* < 0.0001). In contrast, patients with ctDNA negativity status four weeks following surgery had favorable outcomes with an 18-month DFS surpassing 90% for high-risk stage II or stage III CRC, regardless of ACT administration.

An updated analysis of the GALAXY study, presented at the ASCO Gastrointestinal Symposium 2024 and published in *Nature Medicine* [52,53], examined 2240 patients over a median 23-month follow-up. In this analysis, the MRD window (2–10 weeks post surgery, prior to ACT) was evaluated in 2109 patients. Results showed a 2-year DFS of 85.1% in ctDNA-negative patients vs. 20.6% in ctDNA-positive patients (HR 11.99; 95% CI: 10.02–14.35, *p* < 0.0001). Additionally, the 36-month DFS for ctDNA-negative patients was 83.5%, compared to 16.7% in ctDNA-positive patients. ctDNA positivity also corresponded with a lower OS rate, with a 24-month OS of 83.7% in ctDNA-positive patients vs. 98.5% in ctDNA-negative patients (HR: 9.68; 95% CI: 6.33–14.82, *p* < 0.0001). Furthermore, ctDNA positivity was the most significant independent predictor of recurrence, with DFS HR 12.08 (95% CI: 9.56–15.27, *p* < 0.001) and OS HR 9.87 (95% CI: 5.60–17.40, *p* < 0.001). Other negative prognostic factors included BRAF V600E mutation, lymph node positivity, and RAS mutations.

In the surveillance window, ctDNA-positive patients showed a 34-fold increased recurrence risk (HR: 33.56; 95% CI: 26.07–43.20, *p* < 0.0001), with a 24-month DFS of 8.9% vs. 93.2% for ctDNA-negative patients. MRD-positive patients benefited from ACT, reducing recurrence risk by 77% (adjusted HR: 0.23, 95% CI: 0.15–0.35, *p* < 0.0001). Conversely, no significant ACT benefit was observed for MRD-negative patients. Notably, in MRD-positive stage IV patients, ACT improved DFS more significantly for those without prior chemotherapy, whereas no benefit was seen in MRD-negative stage IV patients regardless of neoadjuvant treatment status.

**Figure 4:**
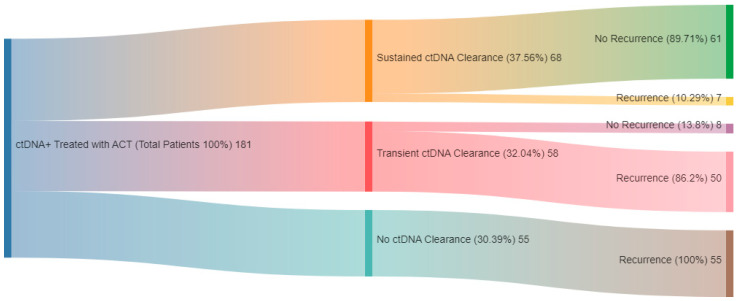
ctDNA clearance rates with adjuvant chemotherapy in MRD-positive patients. Adapted from Nakamura et al. [52,53]

The analysis also highlighted ctDNA clearance as a marker of ACT efficacy (Figure 4). Of the 185 MRD-positive patients receiving ACT, those who achieved ctDNA clearance had improved DFS and OS compared to non-clearers. For instance, a 3-month clearance was associated with a DFS HR of 5.38 (95% CI: 3.59–8.04, *p* < 0.0001) and a 6-month DFS HR of 11.12 (95% CI: 6.09–20.29, *p* < 0.0001). Among MRD-positive patients, those with sustained clearance had the best outcomes, whereas transient or no clearance correlated with significantly poorer DFS and OS (HR for DFS in transient clearance: 19.72, 95% CI: 8.61–45.17, *p* < 0.0001). The study underscores ctDNA’s superior prognostic value over traditional markers like CEA in CRC management, illustrating its potential to guide adjuvant therapy decisions and monitor treatment efficacy through ctDNA clearance status. Interestingly, the study also analyzed spontaneous clearance rates among 151 MRD-positive patients who did not receive ACT. Among these, 105 had subsequent ctDNA results available. Six patients achieved ctDNA clearance; however, three exhibited only transient clearance, with their ctDNA turning positive again during surveillance. All three subsequently experienced clinical recurrence (two with nodal and one with peritoneal recurrence). The remaining three patients showed sustained clearance. Of these, one patient, who had received neoadjuvant treatment, later developed a clinical recurrence in the lung, while the other two remained ctDNA negative and recurrence-free. This resulted in a true spontaneous clearance rate, without clinical recurrence, of 1.9% (2/105). Additionally, among the 2240 patients who underwent ctDNA testing, 124 had positive ctDNA results either during the MRD window or surveillance period but showed no evidence of recurrence, indicating a false-positive rate of approximately 5.5%.

Overall, the GALAXY study provided compelling evidence of the use of ctDNA testing for prognostication of recurrent risk. One of the significant drawbacks of this study, along with the others discussed above, is the observational nature with lack of randomization. However, to investigate the predictive capability of ctDNA further, other clinical trials that are part of CIRCULATE JAPAN are ongoing and will further delineate that role (VEGA and ALTAIR). A recent study published in *Nature* emphasized the prognostic value of tumor-informed ctDNA analysis in patients with LARC [54]. Using the Signatera assay, ctDNA status was evaluated retrospectively in 30 patients post-neoadjuvant therapy (post-NAT) and post surgery. The study also incorporated the Neoadjuvant Rectal (NAR) score to refine recurrence risk prediction. The findings demonstrated that ctDNA positivity post-NAT (HR: 7.82, *p* = 0.001) and post surgery (HR: 19.65, *p* = 0.001) strongly correlated with worse DFS. Patients who cleared ctDNA post-NAT showed significantly better DFS compared to those with persistent ctDNA (HR: 24.7, *p* = 0.001). When combined with the NAR score, ctDNA-positive patients with intermediate or high NAR scores had exceptionally poor DFS (HR: 47.5, *p* < 0.001), while ctDNA-negative patients had better outcomes regardless of their NAR score (HR: 9.8, *p* = 0.0301). These results highlight ctDNA’s potential as a sensitive biomarker for identifying MRD, stratifying recurrence risk, and guiding personalized post-treatment management in LARC.

These studies support the use of ctDNA analysis as a robust prognostic tool that can complement imaging for postsurgical monitoring and recurrence detection in CRC patients.

### 4.2. Studies Using Tumor-Agnostic Approach

Parikh et al. [39] conducted a prospective study using the REVEAL platform by Guardant, which is a tumor-agnostic plasma-only ctDNA assay that integrates genomic alterations with epigenomic cancer signature to detect ctDNA [39]. This was a single-center prospective study that involved 103 patients with stage I-IV CRC treated with curative intent. Periodic plasma samples were obtained preoperatively, 4 weeks prior to surgery, and 4 weeks following completion of ACT. In this study, 19 patients were excluded because they had evidence of disease following surgery or had limited sampling, leaving a total of 84 patients who were evaluated for MRD analysis following completion of definitive therapy. The landmark group included patients who had a plasma sample collected one month after the end of definitive therapy, whether surgery alone or after completing adjuvant therapy. In contrast, the longitudinal analysis group comprised patients who had additional samples taken after their ‘landmark’ point. Investigators classified surveillance draws as those conducted within four months of clinical recurrence. Among 70 evaluable patients, 17 (24%) had detectable ctDNA following the completion of definitive therapy, and 15 of these 17 patients (88%) experienced recurrence within at least one year of follow-up, indicating a specificity of 96.4%. Notably, all 15 patients who had detectable ctDNA one month post therapy relapsed within a median of 162 days following definitive treatment corresponding to a positive predictive value for recurrence of 100% (HR of 11.28; 95% CI, 78.2–100; *p* < 0.0001). On the other hand, a recurrence rate was 24% (12 of 49) in the patients with undetectable ctDNA at landmark analysis. From these findings, the ctDNA landmark analysis demonstrated a sensitivity of 55.6% and a specificity of 100% for predicting disease recurrence. The study further noted that integrating longitudinal ctDNA specimens raised the sensitivity from 55.6% to 69%, while maintaining specificity at 100%. Additionally, incorporating genomic and epidemiological signatures boosted sensitivity even further to 90.9%, without compromising specificity. This study underscores the promise of plasma-only ctDNA assays for MRD detection, though limitations exist, with the low sensitivity at a single time point.

Another study utilizing the Guardant Reveal assay is the COSMOS-CRC-1 study, led by Nakamura et al. [55]. This ongoing multicenter, prospective, non-randomized observational study evaluated Guardant Reveal for detecting MRD in CRC patients. This study included over 1900 plasma samples from 342 patients with resected stage I to III CRC. Samples were collected at specified intervals (day 28 post surgery, then every three and six months) over a follow-up period of up to five years or until clinical recurrence. At the data cutoff, 89% of patients had at least 24 months of follow-up, with a median follow-up duration of 28.4 months. Recurrence occurred in 14% (47/342) of patients, with 83% (39/47) experiencing recurrence at a single site, the most common being pulmonary recurrence. A longitudinal surveillance analysis of 1902 post-surgical samples from 334 patients revealed that ctDNA was detected in at least one sample in 15% of patients (51/334). Breakdown by stage showed ctDNA positivity in 3% of stage I patients (3/115), 13% of stage II (13/99), 26% of stage III (28/108), and 58% of stage IV (7/12). After treatment, 42 patients remained ctDNA positive, with seven clearing ctDNA after chemotherapy, one patient remaining positive only during adjuvant chemotherapy, and one patient with recurrence who had no further post-treatment samples. Patients who stayed ctDNA-negative post treatment had a 24-month RFI of 94.7%, significantly higher than the 40.7% in those who remained ctDNA positive (HR, 16.70; 95% CI, 5.68–49.09; *p* < 0.0001). For patients with recurrence, ctDNA detection had a median lead time of 5.3 months (IQR, 3.0–16.4 months) and extended up to 28.7 months before clinical recurrence, demonstrating ctDNA’s potential to predict recurrence ahead of radiographic confirmation. The assay’s MRD detection sensitivity was 81% for colon cancer and 60% for rectal cancer, the latter likely impacted by lung metastasis, which releases lower ctDNA levels. Sensitivity also varied by recurrence site: 100% for liver metastases, 53% for lung, and 40% for peritoneal metastases. Specificity remained high at 98.2%, indicating a strong capacity to minimize false positives. Postchemotherapy and ctDNA clearance analysis was also conducted in a cohort of 112 stage II or higher CRC patients, paired ctDNA samples were collected at day 28 post surgery and following adjuvant chemotherapy, prior to recurrence. Of these patients, 58% had colon cancer and 42% had rectal cancer. The median duration of chemotherapy was 3.7 months, with post-chemotherapy sampling occurring around 53 days after treatment.

Following chemotherapy, ctDNA was detected in 7.1% (8/112) of patients, who experienced a significantly shorter RFI of 14.9 months compared to ctDNA-negative patients (HR 11.58; *p* < 0.0001). Rates of ctDNA positivity were similar in colon and rectal cancers (7.7% and 6.4%, respectively). The 24-month RFI for ctDNA-negative patients remained high (colon: 90%; rectal: 83.5%). The pattern of ctDNA clearance pre and post chemotherapy provided additional prognostic insight. Patients with consistently negative ctDNA at both time points showed a markedly improved RFI compared to those who only cleared ctDNA after chemotherapy (HR, 0.22; *p* = 0.0041). Notably, 40% of patients with initial ctDNA clearance had recurrence, while only 5% of consistently ctDNA-negative patients did. Additionally, the duration of chemotherapy did not significantly impact ctDNA clearance rates. Further analysis at three months post surgery (in 105 patients) showed similar trends: patients with persistent negative ctDNA had a 24-month RFI above 87%, while those who cleared ctDNA had a 60% RFI versus 0% in patients who did not clear ctDNA (HR, 0.30; *p* = 0.04). One patient who briefly tested positive at three months but later cleared ctDNA did not experience recurrence. By demonstrating a strong link between ctDNA positivity and shorter RFI, this study supports the prognostic value of the Guardant Reveal assay for CRC patients. Guardant Reveal stands out as a promising tool for longitudinal MRD assessment, offering high specificity and consistent performance over extended monitoring periods. The study’s extensive multicenter cohort and long-term sampling contribute to its robustness, but some limitations were noted. Sensitivity challenges arise in detecting certain metastases, such as lung and peritoneal lesions, due to lower ctDNA shedding—a biological limitation affecting all ctDNA assays. Limited data on stage IV patients suggest further studies are needed to evaluate the assay’s efficacy in advanced CRC cases. Extended follow-up may also improve specificity, as some ctDNA-positive cases displayed delayed radiographic confirmation of recurrence. Overall, this study provided important insights into ctDNA-based recurrence prediction, positioning tissue-free assays like Guardant Reveal as accessible, effective tools for MRD monitoring in CRC.

**Table 2:** Completed clinical studies evaluating role of ctDNA as MRD in colorectal cancer.

Author	Number of Subjects and Disease Stage	ctDNA Assay	CtDNA Testing and Surveillance	Median Follow-Up (Months)	Major Outcomes
Tie et al. [16]	230 with stage II colorectal cancer	Safe-SeqS	4–10 weeks postoperative followed by every 3 months for up to 2 years	27	Postoperative ctDNA presence was associated with a 79% recurrence rate at 27 months, compared to 10% for ctDNA-negative patients, and ctDNA detection preceded radiological recurrence by over 5 months—enabling earlier intervention.
Tie et al., 2019 [15]	96 patients with stage III colon cancer	Safe-SeqS	Serial plasma monitoring 4–10 weeks postoperative and within 6 weeks of ACT completion	28.9	Postsurgical cohort, ctDNA positivity was linked to an HR of 3.8 (95% CI: 2.4–21.0, *p* < 0.001), and in the post-ACT group, an HR of 6.8 (95% CI: 11.0–157.0, *p* < 0.001). Three-year RFS for ctDNA-positive vs. ctDNA-negative patients was 30% vs. 77% post-ACT, and 47% vs. 76% post surgery. ctDNA positivity after surgery had a higher HR for recurrence (3.8) than elevated CEA levels (HR 3.4).
Tie et al., 2019 [47]	159 patients with locally advanced rectal cancer	Safe-SeqS	Pretreatment, post treatment CRT, and 4–10 weeks after surgery	24	After CRT, ctDNA positivity was associated with an HR of 6.6 (*p* < 0.001), and post surgery, an HR of 13.0 (*p* < 0.001). Three-year RFS was 33% in ctDNA-positive patients post surgery compared to 87% in ctDNA-negative patients.
Tie et al., 2021 [48]	54 patients with CRC with liver metastasis	SafeSeqS	Samples were collected preoperatively, postoperatively, serially during pre and postoperative chemotherapy, and during follow-up	51	Patients with detectable postoperative ctDNA had significantly lower RFS (HR 6.3; 95% CI: 2.58–15.2; *p* < 0.001) and OS (HR 4.2; 95% CI: 1.5–11.8; *p* < 0.001). Detection of ctDNA at the end of treatment correlated with a 5-year RFS of 0%, compared to 75.6% in those without ctDNA (HR 14.9; 95% CI: 4.94–44.7; *p* < 0.001).
DYNAMIC STUDY Tie et al., 2022 [56]	455 patients with stage II CC	SafeSeqS	4 and 7 weeks post surgery	37	Adjuvant chemotherapy was administered less frequently in the ctDNA-guided group (15%) than in standard management (28%). Two-year RFS was 86.4% in ctDNA-positive patients who received adjuvant therapy, compared to 92.5% in ctDNA-negative patients without it. ctDNA-guided management showed noninferiority to standard management (93.5% vs. 92.4%; absolute difference: 1.1 percentage points; 95% CI: −4.1 to 6.2).
Reinert et al., 2019 [45]	130 patients with stage I to III CRC	Signatera	Preop, postop day 30, and every 3 months for up to 3 years	12.5	30-day postoperative ctDNA-positive patients had a 7-fold higher relapse risk than ctDNA-negative patients (HR 7.2; 95% CI: 2.7–19.0; *p* < 0.001). After completing ACT, ctDNA positivity was linked to a 17-fold higher relapse risk (HR 17.5; 95% CI: 5.4–56.5; *p* < 0.001), and during post-therapy surveillance, ctDNA-positive patients were 40 times more likely to experience recurrence (HR 43.5; 95% CI: 9.8–193.5; *p* < 0.001). Serial ctDNA monitoring detected recurrence up to 16.5 months earlier than imaging (mean: 8.7 months; range: 0.8–16.5 months).
Loupakis et al., 2021 [57]	112 patients with CRC undergoing liver resection	Signatera	Postoperative, at the time of radiologic relapse or last follow-up	10.7	Postsurgical MRD positivity was detected in 54.4% of patients (61/112), with 96.7% of these (59/61) progressing by data cutoff (HR 5.8; 95% CI: 3.5–9.7; *p* < 0.001). MRD positivity was also linked to poorer overall survival (HR 16.0; 95% CI: 3.9–68.0; *p* < 0.001), and ctDNA-MRD status emerged as the strongest prognostic factor for DFS (HR 5.78; 95% CI: 3.34–10.0; *p* < 0.001).
Henriksen et al., 2022 [49]	168 patients with stage III CRC	Signatera	2–4 weeks postop and every 3 months thereafter	35	Postoperative ctDNA detection strongly predicted recurrence (HR 7.0; 95% CI: 3.7–13.5), with an even higher risk immediately post ACT (HR 50.76; 95% CI: 15.4–167). Serial ctDNA monitoring post treatment was similarly predictive (HR 50.80; 95% CI: 14.9–172; *p* < 0.001). Additionally, ctDNA growth rate correlated with survival outcomes (HR 2.7; 95% CI: 1.1–6.7; *p* = 0.039) and indicated recurrence at a median of 9.8 months ahead of radiologic imaging.
GALAXY Kotani et al., 2024 [58]	2240 stages II–IV CRC patients	Signatera	Before surgery, 1-month postoperatively and every 3 months thereafter for 2 years	23	ctDNA positivity during the MRD window was associated with significantly poorer DFS (HR 11.99; *p* < 0.0001) and OS (HR 9.68; *p* < 0.0001). Among patients who recurred, ctDNA positivity correlated with reduced OS (HR 2.71; *p* < 0.0001). MRD-positive patients had consistently shorter DFS across biomarker subsets. Sustained ctDNA clearance after ACT indicated better outcomes compared to transient clearance (24-month DFS: 89.0% vs. 3.3%; 24-month OS: 100.0% vs. 82.3%). True spontaneous clearance without recurrence was observed in only 1.9% (2/105)
Tarazona et al., 2019 [50]	150 patients with stages I to III CC	Tumor-informed ddPCR	Preoperative, 6–8 weeks postoperative, and every 4 months up to 5 years	24.7	Postoperative ctDNA was linked to poorer DFS (HR 6.96; *p* = 0.0001), and remained the only significant predictor after multivariable adjustment (HR 11.64; 95% CI: 3.67–36.88; *p* < 0.001). ctDNA positivity post chemotherapy also indicated reduced DFS (HR 10.02; 95% CI: 9.202–307.3; *p* < 0.0001). ctDNA detection during surveillance predicted recurrence with a median lead time of 11.5 months over radiologic imaging.
McDuff et al., 2021 [51]	29 patients with locally advanced rectal carcinoma	ddPCR	Baseline, preoperative, and postoperative	20	Detectable postoperative ctDNA was linked to poorer RFS (HR 11.56; *p* = 0.007). Among patients with undetectable ctDNA, 13.3% (2/15) experienced recurrence, yielding a negative predictive value of 87%. All patients with detectable ctDNA post surgery recurred, resulting in a positive predictive value of 100%.
Parikh et al., 2021 [39]	103 patients with stages I–IV CRC	Tumor-uninformed assay (REVEAL)	Postoperative, post ACT, and longitudinally in some patients	21.0	All 15 patients with detectable ctDNA recurred (PPV 100%; HR 11.28; *p* < 0.0001). By incorporating serial and surveillance samples (within 4 months of recurrence), sensitivity increased to 69% and 91%. Additionally, integrating epigenomic signatures boosted sensitivity by 25–36% compared to genomic alterations alone.
Overman et al., 2017 [59]	54 patients with stage IV CRC with OM	Guardant Health Reveal	Immediately postoperative	33	Postoperative ctDNA detection was strongly associated with reduced RFS (*p* = 0.002; HR 3.1; 95% CI: 1.7–9.1), with a 2-year RFS of 0% compared to 47% in ctDNA-negative patients. ctDNA detected recurrence at a median of 5.1 months before radiographic evidence.
Lonardi et al. [60]	69 patients with stage IV CRC	Tissue informed personalized assay (FoundationOne	Preoperative, postoperative, post ACT	NR	MRD positivity was linked to lower DFS (HR 4.97; 95% CI: 2.67–9.24; *p* < 0.0001) and OS (HR 27.05; 95% CI: 3.60–203.46; *p* < 0.0001). ctDNA positivity at follow-up significantly reduced DFS (HR 8.78; 95% CI: 3.59–21.49; *p* < 0.0001) and OS (HR 20.06; 95% CI: 2.51–160.25; *p* < 0.0001), with a sensitivity of 69% and specificity of 100%.
Taieb et al. [61]	1345 patients with III CRC	ddPCR	Postoperative, prechemotherapy, 3 months, 6 months	79.2	3-year DFS rate was 66.39% for ctDNA-positive patients versus 76.71% for ctDNA-negative patients (*p* = 0.015). ctDNA was an independent prognostic marker for both DFS (adjusted HR 1.55; 95% CI: 1.13–2.12; *p* = 0.006) and OS (HR 1.65; 95% CI: 1.12–2.43; *p* = 0.011), and remained prognostic in patients treated for 3 months or those with T4 and/or N2 tumors.
Chee [62]	52 patients with oligometastatic CRC	Guardant Reveal	Preoperatively, 3 weeks postoperative, and mutiple structured	12.5	Of the post-ctDNA positive patients, 23 out of 25 recurred (PPV 92%), while 4 out of 20 ctDNA-negative patients recurred (NPV 80%). Median time to radiographic recurrence was 36 weeks for ctDNA-positive patients, compared to not reached for ctDNA-negative patients (HR 7.7; 95% CI: 2.6–22.5; *p* < 0.001).
Shaobo [63]	Stage I–IV CRC 1138 patients	ColonES assay	Preoperatively, postoperatively 1 month, then 3 months	36	Univariate and multivariate analyses identified preoperative ctDNA methylation levels as an independent risk factor for both RFS (HR 2.136; 95% CI: 1.238–3.684; *p* = 0.006) and OS (HR 2.457; 95% CI: 1.398–4.317; *p* = 0.002).
Shaobo [64]	Stage I to III CRC 299 patients		Postoperatively 1 month, then 3 months	36	1-month postoperatively, ctDNA-positive patients were 17.5 times more likely to relapse than ctDNA-negative patients (HR 17.5; 95% CI: 8.9–34.4; *p* < 0.001). Following ACT, ctDNA positivity was linked to significantly shorter recurrence-free survival (HR 13.8; 95% CI: 5.9–32.1; *p* < 0.001). Longitudinally, ctDNA-positive patients had poorer RFS than ctDNA-negative ones (HR 20.6; 95% CI: 9.5–44.9; *p* < 0.001), with ctDNA detecting CRC recurrence at a median of 3.3 months before radiologic confirmation.

Abbreviations: CRC: colorectal cancer, ACT: adjuvant chemotherapy, MRD: minimal residual disease, m: months, HR: hazard ratio, RFS: recurrence-free survival, DFS: disease-free survival, OS: overall survival, CI: confidence interval, NR: not reported, ctDNA: circulating tumor DNA, PPV: positive predictive value, NPV: negative predictive value, ddPCR: droplet digital polymerase chain reaction.

## 5. Role of ctDNA in Evaluating the Efficacy of Adjuvant Therapy in CRC

It is clear from all studies conducted that ctDNA plays a significant prognostic role in MRD assessment. Another potential use of ctDNA is in guiding ACT for postoperative patients. As mentioned earlier, there is heterogeneity associated with TNM staging, impacting which patients will potentially benefit from chemotherapy. One of the challenges with ACT is ensuring that patients who require it receive the maximum benefit while avoiding unnecessary treatment in those who would not benefit, thus reducing chemotherapy-related toxicity. The DYNAMIC study was designed to investigate utilizing the ctDNA-guided approach to determine ACT in patients with stage II colon cancer [56]. This was a phase II, multicenter, randomized controlled trial of biomarker-driven (SafeSeqS assay) adjuvant therapy conducted from August 2015 to August 2019; 455 patients underwent randomization between the ctDNA-guided ACT approach and standard clinicopathologic-guided ACT approach. Plasma specimens were collected for ctDNA analysis from all patients after 4 weeks and 7 weeks from surgery. ctDNA results were available to treating clinicians 8 to 10 weeks after surgery. Patients with positive ctDNA results either at week 4 or 7 received adjuvant single-agent fluoropyrimidine or oxaliplatin-based chemotherapy at the clinician’s discretion, while patients with negative ctDNA results did not receive ACT. The standard management group patients received ACT based on standard clinicopathologic factor criteria. The study had a median follow-up period of 37 months. The primary endpoint was RFS at a 2-year period with a hypothesis of the noninferiority of the ctDNA-guided 2-year-RFS compared to the standard management group and a noninferiority margin of 8.5%. Overall, the study showed that the 2-year RFS was comparable between the two groups (93.5% vs. 92.4% in the ctDNA-guided group and standard management group, respectively). This noninferiority status was maintained after a median follow-up of 59.7 months with a 4-year RFS of 88.3% vs. 87.2% in the ctDNA and standard-of-care group, respectively [65]. The 3-year RFS in ctDNA-negative patients without ACT was 92.5% compared to 86.4% in the ctDNA-positive patients who received ACT (HR, 1.83; 95% Cl, 0.79–4.27). Among ctDNA-negative patients, those with clinically low-risk disease had a higher 3-year RFS than high-risk patients (96.7% vs. 85.1%), suggesting chemotherapy may be unnecessary in patients with low-risk disease and ctDNA-negative results. Additionally, ACT achieved ctDNA clearance in 87.5% (35/40) of postop ctDNA-positive patients correlating with better outcomes (5-year RFS for clearance vs. persistence: 85.2% vs. 20%; HR 15.4; 95% CI, 3.91–61.0; *p* < 0.001). The study had several limitations: while ctDNA-guided therapy reduced chemotherapy use (15% vs. 28%) without impacting DFS, more patients in the ctDNA-guided arm received oxaliplatin (7.5% vs. 2.8%). Additionally, the study’s noninferiority margin of 8.5% is relatively high as it exceeds the typical DFS benefit of adding oxaliplatin to fluoropyrimidine in adjuvant therapy (3% in stage II and 4–6% in stage III [6,7,66,67].

Another study that investigated the role of ctDNA assessment in adjuvant therapy was the COBRA study [68]. The COBRA study was a multicenter, prospective, phase II/III study using plasma-only ctDNA detection as a predictive biomarker to determine adjuvant treatment in resected stage IIA (T3N0) CRC patients. The study randomized 635 patients into the standard of care and the assay-directed therapy groups. In the assay-directed arm, tumor-agnostic assay (Guardant assay) was obtained 14 to 60 days after surgery; if ctDNA was detected, patients received ACT, either CAPOX or mFOLFOX, for 6 months. The primary objective of the study was to compare the rate of ctDNA clearance in ctDNA-positive patients treated with or without ACT following resection. Among 596 patients with available baseline data, ctDNA was detected in 33 patients (5.54%). ctDNA clearance was higher in the surveillance arm (43%; 3/7) compared to the chemotherapy arm (11%; 1/9). Therefore, the study was stopped early for futility based on statistical analysis. The high clearance rate observed in the surveillance group compared to those who received chemotherapy was puzzling and contrary to prior studies. Current studies have reported variations in ctDNA clearance rates in patients with ACT [56,58,69]. As previously highlighted in the GALAXY study, rates of spontaneous clearance without chemotherapy were about 12% compared to 68% with ACT. Another challenge when analyzing these results is the low ctDNA clearance rates in patients who received chemotherapy in the COBRA study compared to what was seen in other studies such as the GALAXY (66.2%) study and DYNAMICS (87.5%) [53,56]. One explanation for the contradictory results is the small sample size of the chemotherapy group, which may have included patients with high ctDNA levels, leading to lower clearance rates. Another limitation of the study was the small sample size of patients with ctDNA positivity and the low incidence of recurrence in patients with stage IIA CRC. Additionally, the study faced challenges with the sensitivity of detecting MRD. The Guardant assay, according to previous studies, demonstrated a sensitivity of around 56% at a single time point and a specificity of 100%, contributing to a higher occurrence of false negatives. This sensitivity issue means that the test may not adequately identify patients who are ctDNA negative but will develop recurrent disease. The low sensitivity poses a challenge in accurately detecting patients with MRD, which can significantly impact clinical decision-making. Overall, the COBRA study was negative and highlighted the need to validate clinical endpoints using relevant ctDNA assay.

An interim analysis of the BESPOKE study [70] was presented at the recent ASCO Gastrointestinal symposium. BESPOKE was a multicenter, prospective, observational study that enrolled 1784 patients between July 2020 and August 2022. ctDNA was assessed and quantified using a personalized, tumor-informed assay in the MRD window (2–12 weeks post-surgery, before ACT), and surveillance window (>2 weeks post ACT or >12 weeks post-surgery if on observation. MRD positivity was higher in stage III (21.87%) than in stage II (6.4%). Patients with MRD-positive status had worse 2-year DFS (29.86%) compared to 91.59% in the MRD-negative group (HR 12.1, 95% CI: 8–18.3, *p* < 0.0001). These findings were consistent across stage II and III CRC. Similar to what was shown in the GALAXY study, MRD-negative patients had comparable outcomes regardless of ACT administration (HR = 1.47, 95% CI: 0.78–2.78, *p* = 0.2316). In contrast, MRD-positive patients had better outcomes when receiving ACT than those who did not (2-year DFS of 42.44% compared to 12.5%; HR = 3.06, 95% CI: 1.43–6.56, *p* = 0.0025). However, it is essential to mention that this was not a randomized study, and selection bias could have contributed to some of these differences. Similar to GALAXY study, patients with sustained clearance had better DFS, while 85% of those with transient clearance experienced molecular recurrence within 15 months. Among roughly 100 patients with disease recurrence, those who underwent ctDNA testing were more likely to receive targeted local therapy. This prospective study further reinforced the prognostic value of ctDNA-based MRD detection and emphasized the utility of ctDNA in guiding oligo-metastatic-directed treatment.

These completed clinical studies have demonstrated the prognostic importance of MRD after curative-intent treatments in the management of CRC. Patients with detectable ctDNA after surgical resection or completion of ACT portend notably poorer RFS outcomes. Current clinical trials are now focusing on therapeutic interventions for patients exhibiting MRD after curative-intent therapies with primary objectives of assessing ctDNA clearance rates and survival outcomes.

## 6. Role of ctDNA in Surveillance

The aim of monitoring patients who are clinically disease-free following curative therapy is to detect recurrences early and intervene promptly to improve clinical outcomes. Current surveillance standards include clinical assessments, regular serum CEA measurements, periodic CT scans, and colonoscopies. However, CEA measurements have low sensitivity and specificity [71,72] and although CT imaging enhances recurrence detection, it has drawbacks such the radiation exposure, reader variability, and high false positive rates [72]. Recent studies suggest that serial ctDNA monitoring offers higher sensitivity and specificity compared to traditional CEA surveillance.

Tie et al. [16] found ctDNA monitoring to be more sensitive than CEA measurements for predicting radiologic recurrence, with 85% of patients being ctDNA-positive at the time of recurrence, compared to only 41% for CEA. Reinert et al. [45] reported ctDNA’ s sensitivity and specificity in identifying relapse as 88% and 98%, respectively, whereas sensitivity and specificity for CEA monitoring in the same population was 69% and 64% respectively. These results highlight ctDNA as a superior tool for detecting disease recurrence compared to conventional CEA monitoring. Furthermore, ctDNA monitoring often identifies recurrence several months before radiologic detection. Overman et al. [59] reported a 5.1-month median lead time between ctDNA detection and radiographic recurrence. Similarly, Tarazona et al. [50] found a median lead time of 11.5 months, and Henriksen et al. [49] reported a median lead time of 9.8 months. Similarly, Reinert et al. [45] noted a mean lead time of 8.7 months. The variation in lead times may be attributed to factors like assay differences and ctDNA shedding. Overall, serial ctDNA assessments outperformed conventional imaging and CEA monitoring, presenting ctDNA as a novel surveillance tool for detecting disease recurrence early and facilitating timely intervention. However, it is still unclear whether early systemic therapy initiation would improve outcomes in a setting of MRD-positivity and negative scans in a surveillance patient. However, a possible advantage could be, more frequent monitoring with shorter interval scans or using alternative/additional imaging techniques, such as MRI or PET-CT. This approach allows for earlier identification of residual disease, potentially enabling timely interventions that can be managed with locoregional treatment strategies.

## 7. ctDNA: Current Applicability

Based on studies such as GALAXY, DYNAMIC, and BESPOKE, ctDNA has shown significant potential in various clinical applications, including monitoring treatment response, prognostication, and detecting recurrence. The DYNAMIC study and others have highlighted the role of ctDNA in MRD detection and assessing recurrence risk. ctDNA effectively identifies MRD in a significant proportion of patients, which might not be detectable through conventional imaging.

These studies have demonstrated the potential future use of ctDNA in guiding adjuvant therapy. Given the high specificity of ctDNA in predicting disease recurrence, future trials should explore whether patients with detectable ctDNA are suitable candidates for intensified adjuvant therapy rather than standard approaches, with the goal of reducing recurrence.

Despite the recent advances with the ctDNA-guided MRD detection, it has significant limitations. The biggest hurdle is whether ctDNA testing can be predictive to determine which patients would most benefit from adjuvant chemotherapy. Further studies are necessary to determine whether ctDNA is a reliable predictive tool for chemotherapy efficacy. Although the GALAXY and BESPOKE studies showed that patients with MRD positive had better DFS with ACT, the non-randomized design of these studies introduces potential bias in the interpretation of results. For instance, clinicians might be more likely to skip chemotherapy for frail, sick MRD-positive patients and administer it to fit, young MRD-positive patients, leading to better outcomes for the latter group. Prospective randomized studies are needed to address this issue. Future research will answer critical questions, particularly regarding the persistence of MRD+ after ACT. The BESPOKE and GALAXY studies, for example, indicated that patients with persistent MRD positive after ACT had worse outcomes. Ongoing trials such as ACT3 [73] and ALTAIR [74] can provide additional information on MRD persistence following chemotherapy. ACT3 (NCT03803553) is assessing the escalation of various treatments, such as chemotherapy with leucovorin, fluorouracil, and irinotecan, or molecularly targeted therapy for patients who are ctDNA-positive after completing chemotherapy for stage III disease. Additionally, ACT3 is exploring the modification of therapy in patients who remain ctDNA positive despite completing standard adjuvant therapy. ALTAIR (NCT04457297) is a randomized, double-blinded, phase III study comparing trifluridine/tipiracil hydrochloride therapy to placebo in resected CRC patients with positive ctDNA after standard adjuvant therapy. Studies like ACT3 and ALTAIR can provide valuable insights into patients with persistent MRD positivity after treatment and the impact of treating MRD compared to reinitiating therapy based on radiographic or other evidence of disease recurrence. Ongoing trials exploring the clinical utility of ctDNA as a marker for MRD in CRC are further highlighted in Table 3.

Additionally, it is essential to highlight the sensitivity challenge involved with ctDNA testing in detecting MRD. The sensitivity of ctDNA testing remains suboptimal, averaging between 40–70% across studies [16,39,48,49,59,62,75]. However, these tests must be more sensitive during the crucial period of 4–10 weeks post operation, as this is the window during which ACT is likely most beneficial. While longitudinal assessments have shown improved positive predictive value (PPV) and sensitivity, better PPV and sensitivity at the landmark analysis are essential to help inform clinical decision-making. Therefore for now, our views and recommendations are consistent with the NCCN guidelines suggesting that there is currently insufficient evidence to recommend the routine use of ctDNA assay outside of a clinical trial to guide adjuvant treatment [4].

A significant challenge in ctDNA testing is the high rate of sample exclusion, often caused by missing or suboptimal samples, which hinders effective testing and data analysis. For example, in the Galaxy study [52], of 2240 patients, approximately 130 were excluded due to missing ctDNA during the MRD window, and 446 were excluded during the surveillance period. Among 336 patients with ctDNA positivity in the MRD window, 89 were excluded at the 3-month mark and 185 at the 6-month mark due to sample limitations. Similarly, in Reinert et al. [45], only 122 of 125 initially enrolled patients were included in the preoperative analysis. By day 30, ctDNA detection rates dropped to 94, and after ACT, only 58 of 77 evaluable patients had ctDNA testing. Likewise, Tie et al. [47] reported drop-offs, often due to missing or inadequate blood samples. These findings highlight significant logistical challenges in plasma collection and high sample failure rates in ctDNA testing, leading to the exclusion of many patients from analyses. Addressing these issues is critical to improving the reliability and scalability of ctDNA testing in clinical trials and routine practice.

Finally, ctDNA has proven to be superior to CEA for surveillance. Multiple studies have demonstrated that ctDNA is more effective in predicting recurrence before it is detectable by imaging. However, the impact of early detection of molecular relapse on the overall survival remains uncertain. Future studies must determine whether early intervention and chemotherapy initiation based on ctDNA-based surveillance can improve overall survival.

**Table 3:** Ongoing trials investigating clinical utility of ctDNA as MRD in colorectal cancer.

Study Identifier	Study Phase	Population	Number	Ct DNA Assay	Study Description	Primary Endpoint
CAREME: NCT05699746 [76]	Phase III	Resected stage I or II CRC	38	MInerVa MRD assay	Arm A: ctdNA-positive treatment arm receives CAPEOX for 6 months. Arm B: ctDNA positive, receives no chemotherapy but on active surveillance.	18-month recurrence-free survival
CORRECT-MRDII: NCT05210283 [77]	Prospective observational	Resected stage II or III CRC	750	Bespoke	Serial ctDNA monitoring after surgery and ACT if received.	Association of post-definitive therapy and pre-recurrence follow-up ctDNA positivity with recurrence-free survival by stage and cancer type
MARIA trial: NCT05219734 [78]	Prospective observational	Stage II–IV with curative intent	Un- known	Invitae personal- ized cancer monitor- ing test		24-month recurrence risk
NRG-G1008; CIRCULATE-NOR-TH AMERICA NCT0517416 [79]	II/III	Stage II and III CC	1912	Signatera	Cohort A: Arm 1—ctDNA negative treated with CAPOX or FOLFOX for 3–6months. Arm 2—ctDNA negative on surveillance with serial ctDNA and no treatment. Cohort B: Arm 3—ctDNA positive treated with CAPOX or FOLFOX for 6 months. Arm 4—ctDNA positive treated with FOLFIRINOX for 6 months.	Time to ctDNA-positive status and disease-free survival

Abbreviations: MRD: minimal residual disease; CC: colon cancer; CRC: colorectal cancer; CAPOX: capecitabine, oxaliplatin; FOLFOX: 5-fluorouracil, leucovorin and oxaliplatin; FOLFIRINOX: 5-fluorouracil, leucovorin, irinotecan and oxaliplatin.

## 8. Conclusions

The current review emphasizes the transformative potential of ctDNA as a precise biomarker for detecting MRD and predicting disease recurrence following curative-intent interventions in CRC. Unlike conventional clinicopathological criteria, ctDNA-based MRD assessment provides enhanced sensitivity and specificity, equipping clinicians with superior insights into tumor dynamics. Once validated in prospective studies, the integration of ctDNA into routine clinical workflows offers the potential to refine risk stratification and optimize surveillance strategies for CRC patients, ultimately facilitating a more personalized approach for adjuvant therapy. Further studies are anticipated to define ctDNA’s role in guiding adjuvant chemotherapy decisions, particularly in the context of therapy escalation or de-escalation, paving the way for a more individualized and evidence-driven approach in the treatment of CRC.

## Figures and Tables

**Table 1:** Comparison of tumor-informed and tumor-agnostic assays for MRD detection.

	Tumor Informed	Tumor Agnostic
Definition	Focuses on mutations associated with a given patient’s tumor	Sequences cfDNA without any information about the patient’s tumor genomics
Genetic Coverage	Limited customized panel of genes based on patient’s tumor	Broad panel of commonly altered genes
Assay used	Cancer-specific targeted panels	Broad NGS panels, whole exome/genome sequencing
Tissue sequencing	Required	Not required
Screening germline, CHIP alterations	Yes	No (it actually filters for CHIP and germline genes)
Turnaround time	Longer period due to tissue sequencing; 3–4 weeks	Shorter, takes 1–2 weeks
Pros	Higher sensitivity for expected variants	Detects novel and unexpected variants
Cons	Potential to miss atypical mutations; higher cost implications	Variable sensitivity and specificity
Applications	Detect MRD Assess treatment response Serial testing for recurrence monitoring	Detect MRD Determine heterogeneity Identify actionable alterations and drivers of resistance Serial testing for disease monitoring

Abbreviations: cfDNA: circulating DNA, NGS: next generation sequencing, MRD: minimal residual disease, CHIP: Clonal hematopoeisis of indeterminate significance.

## Data Availability

Not applicable.
